# Essential functions linked with structural disorder in organisms of minimal genome

**DOI:** 10.1186/s13062-016-0149-y

**Published:** 2016-09-08

**Authors:** Rita Pancsa, Peter Tompa

**Affiliations:** 1 Structural Biology Research Center (SBRC), Flanders Institute for Biotechnology (VIB), Vrije Universiteit Brussel (VUB), 1050 Pleinlaan 2, Brussels, Belgium; 2 Institute of Enzymology, Research Centre for Natural Sciences, Hungarian Academy of Sciences, 1117 Budapest, Magyar Tudósok körútja 2., Budapest, Hungary

**Keywords:** Intrinsically disordered, Structural disorder, Genome reduction, Endosymbiont, Minimal genome, Disorder prediction, Essential function, Genome-reduced bacterium, Chaperone function

## Abstract

**Abstract:**

Intrinsically disordered regions (IDRs) of proteins fulfill important regulatory roles in most organisms. However, the proteins of certain endosymbiont and intracellular pathogenic bacteria with extremely reduced genomes contain disproportionately small amounts of IDRs, consisting almost entirely of folded domains. As their genomes co-evolving with their hosts have been reduced in unrelated lineages, the proteomes of these bacteria represent independently evolved minimal protein sets. We systematically analyzed structural disorder in a representative set of such minimal organisms to see which types of functionally relevant longer IDRs are invariably retained in them. We found that a few characteristic functions are consistently linked with conformational disorder: ribosomal proteins, key components of the protein production machinery, a central coordinator of DNA metabolism and certain housekeeping chaperones seem to strictly rely on structural disorder even in genome-reduced organisms. We propose that these functions correspond to the most essential and probably also the most ancient ones fulfilled by structural disorder in cellular organisms.

**Reviewers:**

This article was reviewed by Michael Gromiha, Zoltan Gaspari and Sandor Pongor.

**Electronic supplementary material:**

The online version of this article (doi:10.1186/s13062-016-0149-y) contains supplementary material, which is available to authorized users.

## Findings

### Minimal bacteria lack structurally disordered regions

Intrinsically disordered proteins (IDPs) and regions (IDRs) of proteins function as ensembles of unfolded conformations [1–4]. They play important regulatory and signaling roles [5], and take central positions in cellular interaction networks [6, 7] as specialists of protein-protein [8–10], protein-RNA [11] as well as protein-DNA interactions [12]. Due to the diverse functional advantages of structural disorder, IDPs/IDRs are widely employed for diverse functional purposes in all three kingdoms of life [13] as well as in viruses [14].

In a previous work, we have confirmed prior assumptions [13, 15] that IDRs are more abundant in eukaryotes than prokaryotes, and highlighted that their abundance not only depends on the complexity of the organisms but also on their lifestyle [16]. Upon analyzing conformational disorder in complete proteomes, it became apparent that in certain endosymbiotic and intercellular pathogenic bacteria that have undergone extreme genome reduction, structural disorder is almost completely lacking [16]. Endosymbiotic bacteria live exclusively within their host, mostly inside specific, dedicated insect cells [17], thus static environmental conditions and a steady supply of nutrients are granted by their host. As they did not need to adapt to environmental changes for hundreds of millions of years [18], their gene sets have been cut to the acceptable minimum [19] by losing entire regulatory and signaling pathways as well and a radical erosion of their remaining proteins, especially at the expense of their IDRs [20]. They have also often undergone functional convergence with other co-resident symbiotic bacteria resulting in metabolic complementarity and co-dependency [18, 19, 21]. Although they have incredibly reduced genomes, they still differ from organelles since they retain relatively robust gene sets that are considered complete enough to enable autonomous life under nutrient-rich, intracellular conditions. Furthermore, their genes are not commonly transferred to the host genome, which is common in the case of organelles [19]. Interestingly, in some of these organisms, high mutation rates coupled with genetic drift makes their proteins susceptible to misfolding, demanding the extensive assistance of chaperones [19]. As these minimal organisms have undergone independent paths of genome reduction in their respective hosts [19], and belong to different phylogenetic clades, their proteomes can be considered as independently evolved minimal protein sets, restricted to proteins of universal importance or of specific function required for the symbiosis [19]. Therefore, with the identification and systematic analysis of IDRs that have been consistently preserved within minimal bacteria, here we uncover and describe the most essential cellular functions relying on structural disorder.

### Identifying long disordered regions within minimal proteomes

We obtained the representative bacterial complete proteomes (<15 % co-membership threshold) from the PIR database (release 2015-04, [22]) and selected the ones with extreme genome reduction (<580 proteins; this threshold warrants that a few Mycoplasma species are included, but they do not overly dominate the data, and that the obtained proteomes are all well-annotated). Luckily, the resulting 13 proteomes represented diverse phylogenetic clades (Table 1).

**Table 1:** Non-ribosomal proteins that retained LDRs in at least three minimal organisms of at least two different bacterial clades

Taxon ID	Clade	Species	rpoB	rpoC	rpoD	infB	prfA	groL	dnaK	ssb	ftsH
1053648 **(115)**	BPB	Cand. Tremblaya princeps PCVAL	✓	✓	ND	Ø	Ø	✓	✓	Ø	Ø
1343077 **(137)**	BPB	Cand. Nasuia deltocephalinicola str. NAS-ALF	ND	ND	ND	Ø	ND	ND	✓	✓	Ø
573234 **(169)**	APB	Hodgkinia cicadicola (strain Dsem)	ND	ND	ND	ND	ND	ND	✓	Ø	Ø
1266371 **(175)**	BPB	Cand. Tremblaya phenacola PAVE	✓	ND	ND	ND	✓	✓	✓	ND	Ø
667013 **(206)**	GPB	Cand. Carsonella ruddii DC	ND	ND	ND	ND	ND	ND	ND	Ø	Ø
871271 **(206)**	BPB	Zinderia insecticola (strain CARI)	ND	ND	ND	ND	ND	ND	ND	✓	Ø
1415657 **(243)**	BAC	endosymbiont of Llaveia axin axin	ND	ND	ND	✓	ND	ND	✓	Ø	✓
1206109 **(273)**	GPB	Cand. Portiera aleyrodidarum BT-B-HRs	ND	ND	ND	✓	ND	✓	✓	Ø	ND
482235 **(448)**	FIR	Phytoplasma mali (strain AT)	ND	ND	ND	ND	ND	ND	✓	ND	✓
347256 **(529)**	TEN	Mycoplasma hominis (strain ATCC 23114)	✓	✓	✓	ND	ND	Ø	✓	✓	✓
515618 **(540)**	GPB	Riesia pediculicola (strain USDA)	ND	ND	✓	ND	ND	✓	✓	✓	ND
107806 **(572)**	GPB	Buchnera aphidicola subsp. A. pisum (strain APS)	ND	✓	✓	✓	✓	✓	✓	✓	ND
1318617 **(572)**	TEN	Cand. Mycoplasma girerdii	ND	ND	ND	ND	✓	Ø	✓	✓	✓
**83333 (4306)**	**GPB**	**Escherichia coli strain K12**	**ND**	**ND**	✔	✔	✔	**ND**	✔	✔	✔
**435590 (3982)**	**BAC**	**Bacteroides vulgatus strain ATCC 8482**	**ND**	**ND**	**ND**	✔	**ND**	**ND**	✔	✔	✔
**224308 (4197)**	**FIR**	**Bacillus subtilis strain 168**	✔	✔	**ND**	✔	✔	✔	✔	✔	**ND**

Taxon ID: the taxon identifier of the organisms with the number of their proteins annotated by UniProt in brackets. Clade: an abbreviation of the corresponding phylogenetic clade (*APB* Alphaproteobacteria, *BPB* Betaproteobacteria, *GPB* Gammaproteobacteria, *BAC* Bacteroidetes, *FIR* Firmicutes, *TEN* Tenericutes). Species and strain information is followed by columns showing information on the 9 proteins with retained LDRs. Different marks mean that i) the given protein was not annotated in the given organism (missing or does not show recognizable sequence homology (Ø)); ii) the protein is not disordered (ND), meaning that the given protein was annotated, but it does not contain an LDR in the given species, and iii) the protein has at least one LDR (✓). Data for non-minimal reference bacteria of three different clades are indicated in the last three lines in bold. A similar table with all corresponding UniProt identifiers indicated in the appropriate cells is available as Additional file 1: Table S2

Structural disorder was predicted for all the associated proteins by IUPred [23, 24], which is a conservative disorder prediction method showing good correspondence [25] with the consensus disorder patterns of MobiDB [26]. The average fraction of disordered residues in the obtained proteomes ranged between 1 and 12 %, with a median of 3.7 % that is extremely low compared to other organisms [16]. Residues with an IUPred score > 0.5 were considered as disordered and all stretches of at least 20 consecutive disordered residues were identified, which will be hitherto referred to as long disordered regions (LDRs). The number of identified LDRs ranged between 1 and 57 including ribosomal proteins, but only 0 to 41 excluding ribosomal proteins (with medians of 17 and 7 regions, respectively).

### Functions relying on structural disorder in minimal proteomes

Ribosomal proteins contained a large fraction of the detected LDRs (See Additional file 1: Table S1). The L2, L4, L15, and L34 protein components of the large ribosomal subunit and S12 and S13 of the small subunit contained LDRs in more than half of the investigated 13 species, while many other subunits also contained LDRs in multiple species of different clades. This is of no surprise, as ribosomal proteins are known to universally rely on structural disorder [27] that enables the formation of tightly packed ribonucleoprotein complexes with ribosomal RNAs.

Table 1 presents all the non-ribosomal proteins (by gene names) that have retained LDRs in at least three minimal proteomes belonging to at least two different bigger prokaryotic clades. The list includes components of the transcription (DNA-dependent RNA polymerase subunits beta and beta’ and sigma factor /rpoB, -C and -D/) and translation (translation initiation factor 2 /infB/, peptide chain release factor 1 /prfA/) machineries, two chaperones (the 60 kDa GroEL /groL/ chaperonin and DnaK), single-stranded DNA-binding protein /ssb/, and the transmembrane ATP-dependent zinc metalloprotease FtsH.

For these 9 proteins, all the retained homologs were aligned by Custal Omega 1.2.2. [28] and the corresponding disorder predictions were projected onto the alignments to see the positional conservation of LDRs.

RNA polymerase subunits (beta, beta’ and sigma, rpoB, rpoC and rpoD, in Table 1) gave the weakest signal. Although they are annotated in all species, they show no sign of disorder in most of the minimal organisms and reference bacteria. For the beta subunit, the few detected LDRs are located internally, while for the beta’, they mainly occur at the C-terminal. The sigma factor has a highly disordered internal loop in two minimal organisms belonging to Gammaproteobacteria, just like in E. coli (see Protein Data Bank (PDB; [29]) ID: 4YG2 [30]), and also in Mycoplasma hominis. Nonetheless, we have failed to find any evidence in the literature for the disordered nature of any particular region in these subunits.

Translation initiation factor 2 (IF2, infB in Table 1) is a GTPase essential for binding initiator transfer RNA to the 30S ribosomal subunit and recruiting the 50S subunit to the initiation complex [31]. The identified homologs show very large deviations in length and disorder pattern within the N-terminal region preceding the GTP-binding domain. Although in the three reference bacteria IF2 has a long, largely disordered N-terminus, the corresponding LDR has only been retained in three minimal organisms. This N-terminal flexible region promotes the joining of the two subunits [32] and makes IF2 largely extended in the complex (see PDB IDs: 3J4J, 3JCN and 3JCJ [33]).

In protein chain release factor 1 (prfA in Table 1), the C-terminal half consistently displays predicted disorder values that fluctuate around the order-disorder threshold. Even though in most minimal organisms this region does not fulfil the criteria of LDRs, the observed tendency is in good agreement with the large domain movements that are required for its interaction with the ribosome and accurate translation termination (see PDB IDs: 1RQ0, 4V7P, 5J4D and 1ZBT [34, 35]).

**Fig. 1:**
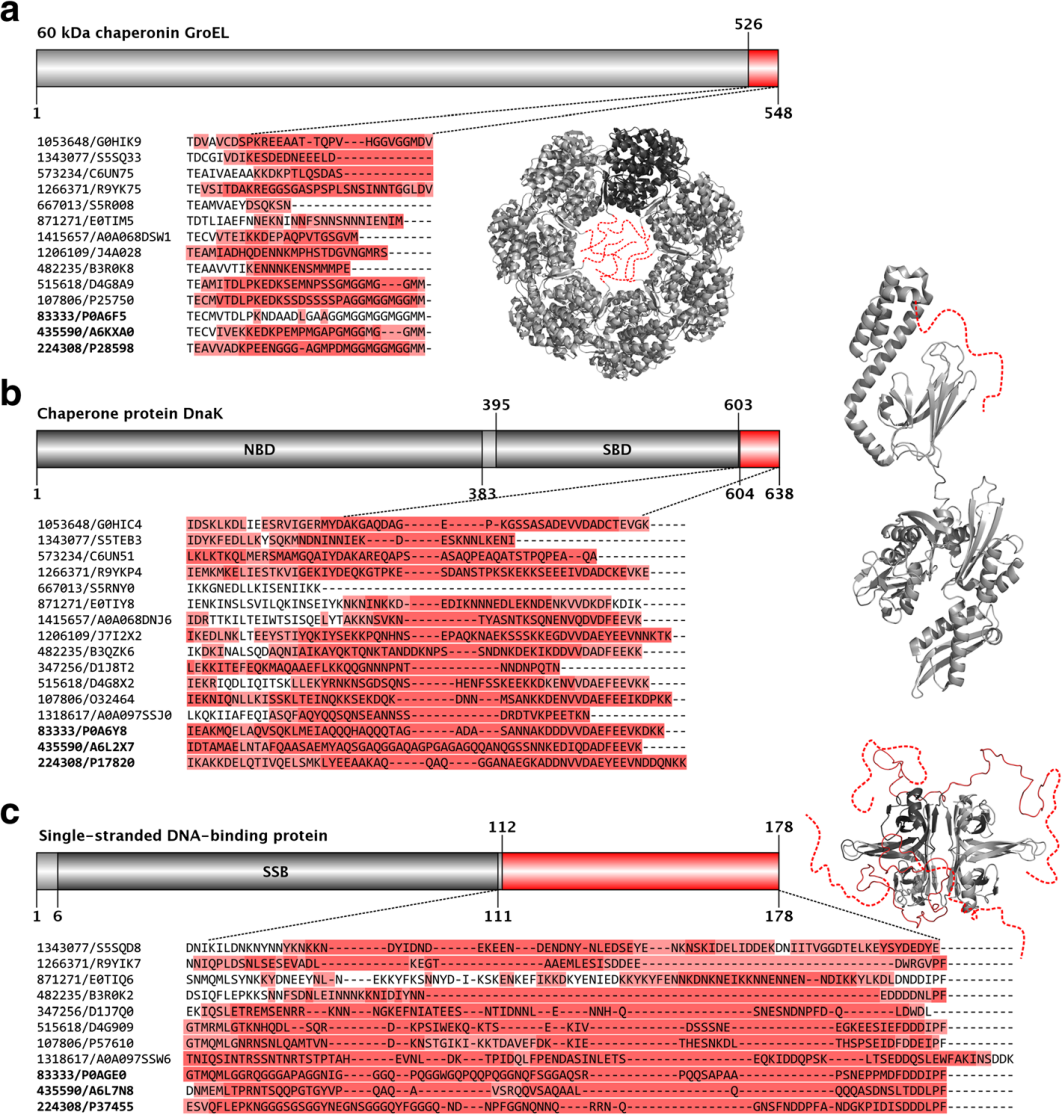
*Conserved disorder in chaperones and hub proteins of minimal organisms*. Domain maps, structures, sequence and disorder conservation are depicted for the three proteins (GroEL (**a**) DnaK (**b**) and SSB (**c**)) with conserved long disordered regions. On the grey domain maps, the residue boundaries of conserved disordered segments (*in red*) and known domains (*in darker grey*) are provided. The red regions of the domain maps complemented by a few residue positions around are also highlighted as Clustal Omega 1.2.2. multiple sequence alignments below the domain maps. The sequences of the minimal and reference organisms are identified by their Taxonomy/UniProt identifiers, and are depicted in the same order as in Table 1. In the alignments the background of the residues are colored according to the corresponding IUPred predictions; residues with a score >0.5 in darker red, while residues with a score between 0.5 and 0.4 in lighter red. The structures of the corresponding E. coli proteins (PDB: 2NWC for GroEL, 2KHO for DnaK and 1QVC for SSB) are also depicted in light grey with the conserved disordered segments marked by red or added as red dashed lines. In the heptameric GroEL and tetrameric SSB structures one chain is depicted by darker grey than the others

Interestingly, chaperones seem to rely on structural disorder the most. For example, the C-terminal tail of GroEL preserved its disordered nature in most minimal and reference species (Fig. 1a). Although the E. coli sequence is not predicted as disordered, this is certainly a misprediction because the respective region is missing from available X-ray structures (Fig. 1a). The highly flexible, hydrophilic tails protrude into the cavity of the ball-shaped chaperonin cage formed by GroEL/GroES subunits and assist the correct folding of proteins [36]. Multiple studies collectively confirmed that truncation of the tail impairs the efficient refolding of substrate proteins [36–39], however its specific molecular role in the chaperonin reaction cycle is still under debate [38, 39].

The C-terminal tail of chaperone protein DnaK (the Hsp70 homolog of bacteria) is the best preserved non-ribosomal LDR in the investigated minimal and reference organisms (Fig. 1b). The tail region enhances chaperone activity and cellular survival upon stress in DnaK [40] and mediates interactions with co-chaperones containing a tetratricopeptide repeat domain in certain Hsp70 proteins [41, 42].

Single stranded DNA binding protein (SSB) is a universally conserved protein that binds ssDNA in a sequence-independent manner, and forms oligomers [43] while sliding on ssDNA [44–46]. The >50 residues long, disordered tails of the monomers remain disordered in the complex [47] and play essential hub roles in DNA replication, recombination and repair by orchestrating the action of an array of genome maintenance proteins [44, 46, 48, 49]. Interestingly, the last nine amino acids provide the docking site for all the known partners [46, 50], while the amino acid composition and length of the conserved LDR (Fig. 1c) is thought to affect ssDNA binding mode preferences [51].

Finally, FtsH is an ATP-dependent zinc metallopeptidase that resides in the membrane and plays an essential role in the quality control of integral membrane proteins [52–54]. It is present in the 7 largest of the selected minimal organisms, with differentially positioned LDRs in four of them. The best-preserved disordered segment resides in its cytoplasmic AAA domain (see PDB IDs: 2R62 and 1IXZ), most probably conferring flexibility on the domain, but there are also compositionally biased LDRs in the N- and C-terminal regions of the protein.

Most of the identified LDRs are genuine disordered regions that fulfill their functions as conformational ensembles and thus are missing from available structures of homologous proteins (see PDB structures: RpoD—4YG2, InfB—3JCN, PrfA—1ZBT, GroEL—2NWC, DnaK—2KHO, SSB—1QVC). When repeating the analysis by requiring a minimum of 30 consecutive residues for an LDR, we could still identify 7 ribosomal proteins (Additional file 1: Table S3) and 4 other proteins (Additional file 1: Table S4; RpoD, InfB, DnaK, and SSB) that retained LDRs in at least three minimal organisms from at least two clades.

We have also repeated the whole analysis with the ESpritz X-ray prediction method [55] using its conservative version (5 % false positive prediction rate). The observed tendencies were largely preserved. Out of the 18 ribosomal proteins displaying conserved LDRs by IUPred, three did not show conserved LDRs with ESpritz, while another four only fulfilled the criteria with ESpritz (Additional file 2: Table S5). Regarding other proteins, with the exception of PrfA, all identified proteins listed in Table 1 displayed conserved LDRs also by ESpritz in the same regions as predicted by IUPred, while the replicative DNA helicase /dnaB/, protein GrpE /grpE/ and ribonuclease 3 /rnc/ were newly identified (Additional file 2: Table S6). We have checked the IUPred predictions of these novel proteins and confirm that they have just missed the criteria for conserved LDRs by displaying LDRs in one or two minimal organisms. When requiring a minimum of 30 consecutive residues for an LDR, 7 ribosomal proteins (Additional file 2: Table S7) and 5 other proteins were retained, namely RpoC, InfB, DnaK, SSB and GrpE (Additional file 2: Table S8).

## Discussion

The positional conservation of certain LDRs within minimal organisms of different bacterial lineages implies that structural disorder is essential for certain functions in the cell. It has always been thought to be essential in the functioning of the ribosome, not only because many ribosomal proteins contain disordered segments [27], but also because it facilitates the critical domain movements of important translation initiation and termination factors. Structural disorder has also been implicated in the functioning of RNA- and protein chaperones [56], and chaperones have been suggested to be one of the most disordered functional protein class in the proteome. Besides classical chaperones [36, 40], fully disordered stress proteins also can have chaperone activity [57], and thus the observation that two chaperones and other proteins playing a role in protein homeostasis (GrpE and FtsH) have retained their high level of structural disorder in organisms of minimal genome, fits strongly with this trend.

DNA/RNA-binding proteins are also among functional classes that abound in structural disorder, with disorder contributing to DNA binding, RNA binding and protein-protein interactions, such as is transcription factors [11, 58]. In full agreement, we found a conspicuous enrichment for structural disorder in transcription/translation-related proteins in organisms of reduced genome. In addition, structural disorder is also central to proteins of hub function, due to structural adaptability accompanying induced folding, enabling IDPs to mediate multiple interactions; in addition, short interaction motifs enable very high functional density of IDRs [6]. This is manifested in the structural disorder of the C-terminal tail of SSB, probably the most universally conserved disordered hub that mediates specific interactions with different binding partners through a single short linear interaction motif [48], thereby orchestrating the different steps of DNA processing.

In all, the general functional feature of IDPs/IDRs that emerges from our study is that the most essential functions in which long IDRs are indispensable are the ones in which they mediate or regulate interactions with other macromolecular partners, e.g. RNA or proteins. In these, specific recognition has to be combined with the lack of strict sequence or surface characteristics. Typical examples are chaperones, in which IDRs contribute to recognizing ill-defined misfolded states of unrelated proteins, RNA-binding proteins in which IDRs bind distinct RNAs of variable secondary and tertiary structural features, and DNA-binding proteins, in which disordered regions are either directly involved in contacting DNA [58] or mediate domain movements involved in recognizing ssDNA, irrespective of its sequence. The advantage provided by structural adaptability of IDRs in these functions is unparalleled, and thus we conclude that these functions are amongst the most essential and most ancient functional roles fulfilled by structurally disordered regions in cellular organisms.

## Reviewers’ comments

### Reviewer 1: Michael Gromiha

**Reviewer comments:**

In this work, the authors systematically analyzed structural disorder in a set of minimal organisms and showed that few characteristic functions are linked with conformational disorder. Further, they suggested that these functions correspond to the most essential ones fulfilled by structural disorder in cellular organisms. The analysis has been carried out extensively with specific examples to RNA polymerase, chaperone protein, single stranded DNA binding proteins, DnaK and so on. The work is interesting and the data provide new insights.

The following comments may be addressed for improvements.

1. The results obtained with negative dataset may be discussed.

Authors’ response: *We thank the reviewer for appreciating our work and his suggestions for improvements. We are not entirely sure what the reviewer means by negative dataset. Since the investigated proteomes are extremely minimalized, which affect both the number and the length of proteins, in some of them even the otherwise essential proteins listed in* Table 1*are missing. Also, in many of them the disordered regions either disappeared or shortened to an extent that they do not fulfil our criteria of LDRs (*Fig. 1*indicates that even though in some cases the disordered regions are shorter, they are mostly preserved). Now we added orthologs from reference bacteria of different phylogenetic groups to address if the identified regions are generally disordered or only due to the minimalistic nature of the investigated species. We find that many are also disordered in the reference proteomes. Now we also validate our results by using another method for disorder prediction.*

2. The threshold value of 580 proteins for selecting the proteins with extreme genome reduction may be justified.

Authors’ response*: We have chosen the threshold of 580 for proteome size because above this threshold the obtained proteome set would have been too much biased towards Mycoplasma species. With this threshold we ensured that only two Mycoplasmas are selected, but obligate endosymbionts of different phylogenetic groups are still well represented. Also, somewhat above this threshold there are non-Mycoplasma proteomes in which the proteins were not annotated, only numbered and hence they could not have been used for this analysis.*

### Reviewer 2: Zoltan Gaspari

**Reviewer comments:**

This paper describes some important findings about the role of intrinsic protein disorder in minimal genomes. Its original and can be of interest for researchers working in the respective field. Although I think that the work contains novel findings, the volume of the data processed and the novel information provided is a bit limited. I think that the amount of sequences analyzed makes a more detailed study possible and I make some recommendations for this below that the authors might consider to improve the manuscript.

- The authors used one prediction algorithm (IUPred), one threshold (0.5) for classifying residues and one (20 aa) for identifying long regions. Where such necessarily subjective choices should be made, it can be important to prove the robustness of the main conclusions by repeating the analysis with some parameters varied. Can the authors provide such considerations?

Authors’ response*: We thank the reviewer for appreciating our work and his suggestions for improvements. IUPred is a very widely used and trusted method that shows good correspondence with consensus predictions obtained based on many different methods. In many of the previous analyses we and others have repeated the predictions with different methods and the identified tendencies were always preserved. This is why we thought one trusted method should be enough to point out such tendencies. Also, some of the methods that can be locally used on complete proteomes, for example VSL2B is known to predict very similar patterns but with elevated absolute values, meaning that it usually overestimates the number and extent of disordered regions compared to consensus disorder patterns. In this analysis overprediction is definitely not desired because we wanted to find the set of protein regions that consistently preserve their disordered nature. In our view it is better to obtain a relatively restricted but stable set of regions, than getting a larger set that is diluted with false positive cases. Now we repeated the analysis using another prediction method, ESpritz X-ray, and we find very similar tendencies as described in the last paragraph of the findings section.*

*The published threshold value for IUPred that discriminated folded and disordered regions the best is 0.5; this is not a value that we considered to change. In* Fig. 1*, however, we have also highlighted residues with a prediction score >0.4 but <0.5 to show that disorder scores often do not drop very steeply and thus the residues surrounding or intervening predicted disordered regions usually receive predicted scores implying high flexibility.*

*We have now tried to look for even longer regions of 30 consecutive residues. Only 7 ribosomal and 4 non-ribosomal proteins retained LDRs of at least 30 consecutive residues in at least three minimal organisms from at least two of the represented bacterial clades. We added two* Additional file 1: Tables S3 and S4 *to show the results with this parameter, and also describe those in the manuscript.*

*We did not want to look for regions <20 residues because those cannot be considered as LDRs (at least we do not know of any analysis in the ID literature where <20 residues long regions were considered as LDRs). 20 residues thus seemed as the ideal choice. The C-terminal region of GroEL, for example, is just between 15 and 25 residues in most organisms regardless of proteome size, which is most probably restricted by the size of the interior cavity. Multiple experiments with deletion mutants for this region demonstrate that true ensemble-like LDRs of around 20 residues can fulfil crucial functions (see literature references* [36–39]*).*

- It could be of interest to analyze some of the protein families with LDR regions in a bit more detail, including sequences from organisms with non-minimal genomes. There might be interesting patterns in the presence/absence of LDRs that are only apparent on a larger data set.

Authors’ response*: We agree. We extended the analysis,* Table 1*, all additional tables and the alignments of* Fig. 1*with orthologs from non-minimal reference bacteria as explained below.*

- In general, the study could benefit from using some “reference organisms” with non-minimal genome from all investigated groups. It can be of interest whether any feature might be associated with being in a minimal genome.

Authors’ response*: We agree. We extended the analysis,* Table 1*, all additional tables and the alignments of* Fig. 1*with some reference organisms, namely Escherichia coli (strain K12) representing Proteobacteria, Bacteroides vulgatus (strain ATCC 8482) representing Bacteroidetes, and Bacillus subtilis (strain 168) representing the Firmicutes clade. From Tenericutes we did not include a reference proteome because those are best represented by Mycoplasmas, which are included in the dataset anyways. However, we did not accept further protein hits that only show disorder in the reference proteomes because we were explicitly interested in proteins/protein regions that preserve their disordered nature after severe genome minimisation. We only use the reference proteomes to demonstrate that the identified LDRs are also mostly present in those and are thus not a consequence of genome minimization, or the associated fast evolutionary changes and instability of the proteins.*

- The authors might want to comment on whether all the identified LDRs are ‘genuine’ disordered regions and not coiled coils or other segments commonly predicted to be disordered.

Authors’ response*: We do not know about the sparsely detected regions within polymerase subunits b and b’ and FtsH, because those are not consistently disordered and not mentioned in the literature as such. However, the other regions that we identified, the C-terminal tails of DnaK, GroEL, a linker within RpoD and the N-terminus of GrpE and InfB, as well as the tail region of SSB were checked and they are all ‘genuine’ disordered regions, which correspond to missing regions in the respective PDB structures, as now explained in the manuscript.*

- It is not described how the authors identified orthologs as gene/protein names might not be conclusive. Kindly comment on this as there are many missing homologs/orthologs indicated in Table 1.

Authors’ response*: We actually identified orthologs based on gene and protein names, because we found that in case of these minimal organisms and the associated crucial proteins those were conclusive. When absolutely crucial proteins were missing we were also trying to identify those with blast, but we could never find them. For instance we were surprised to see that GroEL was missing in the two Mycoplasmas that are not even among the smallest proteomes, however we could not find any sequence resembling GroEL and finally found publications stating that in many Mycoplasmas the GroEL-GroES system is completely missing (Wong P and Houry WA, 2004). So the missing homologs/orthologs in* Table 1*are really missing as a consequence of genome minimization and not due to misannotation.*

- The authors might want to comment on the proteins with LDRs found in only one of the investigated proteomes. It can be of interest whether these proteins are in any way associated with being located in a minimal genome.

Authors’ response*: This is an interesting suggestion but in our view this would be out of the scope of this discovery note. We did not identify anything that seemed specific for minimal genomes, however they have surprisingly many putative genes/proteins regarding their minimalistic nature. Some of those are disordered. Since several works suggest that their proteins need the extensive assistance of chaperones, we were guessing that among those putative proteins there might be disordered chaperones, but we cannot prove this assumption. Also, we are highly restricted with both text length and number of display items, so we would like to stick to the original idea that is, looking for regions that are consistently disordered in different phylogenetic groups after extensive genome reduction.*

- Page 5, line 39: “translation initioation factor” is misspelled.

Authors’ response: *We thank for this remark, we corrected the mistake.*

- The two additional xls files could be combined to a single one with the data in two tabs.

Authors’ response*: We thank for this remark, now all the Additional Tables with IUPred data are arranged as tabs of a single excel file and those with ESpritz are collected in another.*

### Reviewer 3: Sandor Pongor

**Reviewer comments:**

The manuscript by Pancsa and Tompa highlights the fact that in organisms with a minimal genome, essential protein functions are linked with structural disorder. The ms is well written and understandable. The figures are clear.

I feel that the message could be made more succinct by emphasizing a few aspects that may not be immediately clear to wider audiences. For instance, i) Why was the set of 13 proteomes selected? Is the finding—i.e. the set of proteins found—sensitive to the selection? The NCBI list of complete (annotated) genomes includes over 80 endosymbionts. In more detail, the Mazumder dataset is optimized for sequence similarity as well bibliographic criteria in the context of all proteomes, while taxonomic coverage within the group of the selected (minimal genome) organisms may be more relevant to this work, at least according to this reviewer. The original paper of Mazumder et al states that CMT55 is superior to other dbase distributions in this respect ü can the author explain why they chose the CMT15 dataset?

Authors’ response*: We thank the reviewer for appreciating our work and his suggestions for improvements. We have chosen the CMT15 dataset because that contains well-annotated species, whose phylogenetic group is assigned. Although this dataset is smaller than the CMT55, the quality of annotations is much better. For Bacteria, the CMT55 dataset is identical to the list of UniProt reference proteomes that currently contains 4159 proteomes (UniProt 08_2016). Back than in 2011 when the Mazumder paper was published they had altogether only 637 proteomes in the CMT55 dataset, while now it is over 5000. The expansion of the sequence space is so fast that annotation procedures can clearly not catch up any more. So, although there are much more proteomes in CMT55, many of those are not well annotated, many come from environmental samples, there are several species represented from the same genus, so it is quite redundant, and there are many unclassified species whose phylogenetic group is not known (for example there are multiple reference proteomes with GW numbers termed as Parcubacteria group bacterium or Microgenomates group bacterium). Also, we have found several mistakes that would potentially affect our dataset, for instance under the UniProt code UP000064377 there is a Salmonella enterica subsp. enterica serovar Enteritidis str. LA5 species assigned as a reference proteome with only 102 proteins. It was clear that this annotation cannot be correct, since Salmonella enterica species usually have huge genomes and proteomes with >5000 proteins. We have checked this entry and it turned out that it contains only the proteins of a respective plasmid. Although there are mistakes that are easy to identify and filter out, there are also less shouting annotation mistakes that would not necessarily pop up in the automated data analysis pipeline used here, so we decided to use the smaller but better annotated, more trustworthy CMT15 dataset. For the proteomes that we use, the genes and proteins are also well annotated, so we can rely on the annotated gene and protein names, while for many reference proteomes of the CMT55 dataset they are only numbered with no information on the function of the protein whatsoever. Lastly, if using a considerably larger dataset we could not show the corresponding data table in the manuscript, neither to depict the complete alignments of the orthologs.*

ii) Are the orthologues of the identified proteins found in non-reduced genomes also disordered?

Authors’ response: *For the polymerase subunits we did not find any literature evidence on conserved disordered regions, but for DnaK, DnaJ, GroEL, GrpE, SSB, translation initiation factor 2 (infB) and Peptide chain release factor 1 (prfA) there are analyses in the literature and available protein structures supporting that the respective regions are disordered in orthologs from non-reduced genomes. Now we included three representative reference bacterial proteomes into the analysis to show the disorder status of the identified regions in those and mention the corresponding protein structures.*

iii) The identification of disordered proteins relies on the prediction of long disordered stretches. Does the length threshold and the selection of the prediction program influence the findings? Would a different prediction method give different functional predictions?

Authors’ response: *We have already answered similar questions for Zoltan Gaspari above. The length definitely influences the findings. With a minimum LDR length of 30 consecutive residues, we found less conserved disordered regions, but 7 ribosomal proteins and 4 other proteins still retained LDRs in more than one bacterial clade (see newly added* Additional file 1: Table S3 and S4*.*

*Twenty residues seemed as the ideal choice for an LDR. We did not want to look for regions <20 residues because those cannot be considered as LDRs.*

*Now we repeated the analysis using another conservative prediction method, ESpritz X-ray, and we find very similar tendencies. Please see the new paragraph before the Discussion section.*

iii) Can one assign statistical significance to the findings, for instance by simply repeating the predictions with a series of subsets of the selected proteomes?

Authors’ response*: We could maybe assign statistical significance but we do not think it is necessary. The average fraction of disordered residues in these proteomes ranges between 1 and 12 %, with a median of 3.7 % that is extremely low compared to other organisms (Pancsa and Tompa, 2012). The number of identified LDRs was between 1 and 57 in the 13 minimal proteomes including ribosomal proteins, but only 0 to 41 excluding ribosomal proteins (with medians of 17 and 7 regions, respectively). The chance to repeatedly pick the same 20 residues long protein segment just by chance (without assuming the evolutionary conservation of disorder in those regions) is negligibly low. We do not think we must force statistics on that, especially that we are bound by the strict length limitations of the discovery note format. It is clear that most of the regions identified here (except for those in the polymerase subunits and FtsH that appeared in different regions of the orthologous proteins) represent evolutionarily conserved disordered regions that do not only pop up due to prediction or annotation mistakes. Now we show that the respective regions are identified independently from the prediction method used and that they are also mostly disordered in non-minimal reference bacteria from diverse clades.*

## Additional files

Additional file 1: Table S1-S4. Ribosomal and non-ribosomal proteins of minimal and reference bacteria with conserved disordered regions of at least 20 or 30 residues predicted by IUPred. (XLS 42 kb) Additional file 2: Table S5-S8. Ribosomal and non-ribosomal proteins of minimal and reference bacteria with conserved disordered regions of at least 20 or 30 residues predicted by ESpritz X-ray. (XLS 51 kb)

## Abbreviations

ATP: Adenosine triphosphate
GTP: Guanosine triphosphate
Hsp70: Seat shock protein 70
IDP: Intrinsically disordered protein
IDR: Intrinsically disordered region
LDR: Long disordered region
NMR: Nuclear magnetic resonance
PDB: Protein Data Bank
PIR: Protein information resource
SSB: Single stranded DNA binding protein
ssDNA: Single stranded DNA
